# Clinical relevance of plasma-derived exosomal long non-coding RNAs (lncRNAs) CCAT1 and XIST in colorectal cancer patients

**DOI:** 10.22099/mbrc.2025.51654.2061

**Published:** 2025

**Authors:** Fatemeh Dana, Soleiman Mahjoub, Zahra Shokati Eshkiki, Abolfazl Namazi, Seidamir Pasha Tabaeian, Abolfazl Akbari

**Affiliations:** 1 Department of Clinical Biochemistry, School of Medicine, Babol University of Medical Sciences, Babol, Iran; 2 Student Research Committee, Babol University of Medical Sciences, Babol, Iran; 3 Cellular and Molecular Biology Research Center, Health Research Institute, Babol University of Medical Sciences, Babol, Iran; 4 Alimentary Tract Research Center, Clinical Sciences Research Institute, Imam Khomeini Hospital, Ahvaz Jundishapur University of Medical Sciences, Ahvaz, Iran; 5 Department of Internal Medicine, School of Medicine, Iran University of Medical Sciences, Tehran, Iran; 6 Colorectal Research Center, Iran University of Medical Sciences, Tehran, Iran

**Keywords:** Colorectal cancer, Long non-coding RNAs, LncRNA, XIST, CCAT1, Exosome

## Abstract

The expression level of exosomal long non-coding RNAs (lncRNAs) can be relevant for clinical diagnostic approaches. The object of our study was to evaluate the differential expression of lncRNAs colon cancer associated transcript 1 (CCAT1) and X-inactive specific transcript (XIST) in plasma exosomes of colorectal cancer (CRC) patients and investigate their potential as clinical biomarkers. In a case-control study, 62 CRC patients and 62 healthy persons were studied. Plasma exosomes were isolated by a centrifugation approach and were characterized by microscopy and western blotting. After RNA extraction and cDNA synthesis, using real-time PCR technique, the relative expression of lncRNAs was evaluated. The expression levels of lncRNA CCAT1, but not XIST, were meaningfully increased in the plasma-derived exosomes of CRC patients compared to non-cancer individuals (p= 0.001, 0.083 respectively). Further analyses revealed that the expression levels of exosomal lncRNA CCAT1 were associated with the lymphovascular invasion and tumor differentiation (p<0.05). ROC curve analysis documented a diagnostic power for lncRNA CCAT1 in CRC with a sensitivity of 79% and a specificity of 80% with an optimal cutoff point 6.5, with an area under curve (AUC)=86% and p<0.0001. Also, lncRNA XIST revealed a sensitivity of 62% and a specificity of 61% with a cutoff point 2.4, with an AUC=65%. Our findings indicated the potential of plasma-derived exosomal lncRNA CCAT1 as a non-invasive clinical indicator for the diagnosis of CRC patients.

## INTRODUCTION

Colorectal cancer (CRC), as one of the most common types of gastrointestinal malignancies, is characterized by high morbidity and mortality worldwide. Because of disease nature, heterogeneity and diagnosis at late-stage, the 5-year the survival rate of CRC patients is usually low [1-4]. Although advanced screening approaches have improved overall survival of CRC patients, these methods still have some limitations such as invasiveness and high cost. Therefore, identification of helpful biomarkers for screening, early detection, and efficient therapy of CRC are very useful [5-8]. In recent decades, liquid biopsy-based non-invasive approaches for detection of cell-free circulating nucleic acids, including key tumor suppressor, oncogenes and non-coding RNAs (ncRNA) have become an interesting biosources to identify clinical biomarkers. In this regard, circulating cancer‐related components, including extracellular vesicles (EVs), and tumor‐educated platelets has increasingly attracted a lot of attention in diagnostic oncology [9-11].

Exosomes are a group of small membranous EVs with 30-150 nm in diameters that derived from nuclear endosomes and released from the cells in an endocytic manner. Exosomes make possible intercellular communication through transporting various signaling effectors, including nucleic acids and proteins [12, 13]. Hence, exosomes play a role as key mediators of transferring cancer-related molecular effectors driving the tumor microenvironment (TME) and heterogeneity. 

Considering the main role of exosomes in determining the biological processes of tumor cells, they have become as promising biological and clinical source for identification of clinical biomarkers in a number of pathological statuses, including cancer [14-16]. These EVs contain a range of biological components, including proteins, lipids and nucleic acids (DNA, mRNA and non-coding RNAs). Exosomal non-coding RNAs (ncRNAs) are gaining increasingly attention as one of the attractive sources of biological elements; their signatures have indicated as possible clinical biomarkers for the cancer detection [17-19].

Long non-coding RNAs (lncRNAs) are characterized as a group of functional ncRNAs over 200 nucleotides in length with critical roles in cellular processing, including cell proliferation, differentiation and apoptosis. Therefore, these non-coding molecules are contributed in cancer initiation and invasion. Emerging evidences have revealed an association between lncRNAs and tumor clinicopathological features, indicating clinical significance of them as promising clinical hallmarks for CRC [20-23]. 

Moreover, the expression levels of exosomal lncRNAs have been documented to be correlated with the tumor stage of cancer patients. Growing studies have demonstrated that tumor-derived exosomal lncRNAs release into and is detectable in liquid biopsies in a high quantity. Exosomal lncRNAs also are protected from RNases and their integrity and function are unchanged. Hence, the tumor-derived exosomal lncRNAs may be right targets for promising clinical biomarkers owing to stability, accessibility and being convenience detectable [24-26]. Based on previous findings, the expression level of these molecules in cell-free liquid biopsies such as plasma can be relevant for clinical diagnostic approaches. 

However, the involvement of potential lncRNAs in diagnostic and therapeutic aspects of colorectal oncology has not yet been well studied and needs further investigation. In this regard, several investigations on circulating specimens, including blood-based biosources have revealed an association with dysregulation of lncRNAs colon cancer associated transcript 1 (CCAT1) and X-inactive specific transcript (XIST) with clinical features of CRC. The findings have indicated potential significance of lncRNAs XIST and CCAT1 as clinical biomarkers in various malignancies [27-31]. Our purpose was to examine the expression pattern and clinical significance of plasma-derived exosomal lncRNAs CCAT1 and lncRNA XIST in colorectal cancer patients.

## MATERIALS AND METHODS

### Patients and blood sample collection:

After completing the informed consent by contributors, total whole blood specimens were collected from 62 CRC patients who were diagnosed as CRC with no chemotherapy and 62 healthy individuals with no malignancy or any diseases referred to Rasool Akram Hospital, Tehran, Iran between July 2022 and June 2023. The inclusion criteria were the confirmation of colorectal malignancy by a surgeon during the colonoscopy, and pathological assays. Exclusion criteria were the benign intestinal polyps, consumption of any chemotherapy drugs, and the presence of other malignancies. Demographic information and clinicopathological characterizations of all CRC patients, including age, sex, tumor size, tumor stage, tissue differentiation, lymphovascular invasion (LVI) were collected from medical records (Supplementary Table S1). The study was permitted by the Ethical Committee of Iran University of Medical Sciences (Ethical Code: IR.IUMS.REC.1399.635).

### Isolation of exosomes from plasma:

7 ml whole blood was gathered from individuals in EDTA-2K vacutainer tubes. Plasma was separated from blood cells using Ficoll gradient centrifugation as previously described [22]. Ribo^TM^ Exosome Isolation Reagent (Ribobio, China) was applied for plasma exosome separation according to the manufacturer’s guidelines. Briefly, centrifugation of plasma samples was completed at 2100 × *g* for 14 min to remove any cells or debris. Then, simplified plasma (3 ml) was transferred to another microtube containing 1 ml Ribo Exosome Isolation Reagent and incubated overnight at 4°C. Afterward, a centrifugation procedure was completed at 15000 × *g* for 90 seconds. The supernatant containing exosomes was transferred to a sterile microtube and resuspended in 100 μl 1X phosphate-buffered saline (PBS).

### Using DLS for evaluating the quality of exosomes:

 For nanoparticle analysis, the dynamic light scattering (DLS) was used to examine particles in the solution. The isolated exosomes were suspended in a volume of 200 microliters. The solution containing exosomes was shaken and the size of the exosomal particles was evaluated using DSL. 

### Exosome characterization by transmission electron microscopy:

About 0.7 mg/ml exosomes speckled onto a glow-discharged copper grid on the filter paper were dried for 15 min. Afterward, exosome staining was completed in a 1% aqueous solution of phosphotungstic acid for 8 min. For drying stained exosomes, the construction was exposed for 20 min to an infrared lamp. Then, exosomes were evaluated under transmission electron microscopy at 100 keV. 

### Western blotting technique:

The isolated exosomes were suspended in radioimmunoprecipitation assay (RIPA) lysis buffer on ice. The quantification of protein concentration was evaluated by a Protein Assay Kit (Parstous, Iran). The specific protein markers were visualized using sodium dodecyl sulfate–polyacrylamide gel electrophoresis (SDS-PAGE) and then western blotting. The diluted protein with loading buffer incubated at 100˚C for 7 min. 25 µg protein was placed on and electrophoresed using sodium dodecyl-sulfate polyacrylamide gel electrophoresis (SDS-PAGE). Then, the electrophoresed proteins were transported to polyvinylidene fluoride (PVDF) membranes, blocked in 4.5 % non-fat milk for 60 min and incubated overnight at 4˚C with primary mouse anti-CD63, anti-CD9 and β-Actin. Primary antibodies anti-CD63 and -CD9 (Abcam, Cambridge, UK ) and anti-β-actin (Abcam, Cambridge, UK) were used.

### RNA extraction from exosomes and cDNA synthesis:

Total RNA was extracted from isolated exosomes using TRIzol Solution (Invitrogen , Paisley, UK). Briefly, TRIzol reagent was added to the suspended source containing exosomes. After addition of chloroform, remained RNA in the aqueous phase was recuperated by precipitation with isopropanol. The RNA was re-dissolved in nuclease-free water. The extracted RNA was stored for future research applications in -80°C. Total RNAs were examined using electrophoresis and NanoDrop 2000. Overall, cDNA synthesis was completed by using 2 µg of total RNA by the PrimeScripst™ RT reagent Kit (Takara, Japan) in line with manufacturer guidelines. The synthesized cDNA was stored at -20°C for following use.

### Real-time PCR for lncRNA expression analysis:

The expression analysis of lncRNAs was completed by quantitative real-time PCR using SYBR^®^Premix Ex Taq™ II kit (Takara, Japan) on 7500 Real-Time PCR System (Applied Biosystems, CA, USA). The expression of lncRNAs XIST and CCAT1 were normalized against β-Actin as a housekeeping gene. Based on the means of ΔCT for tumor and healthy individuals, the relative expression of lncRNAs was examined and the fold change was analyzed using the 2^−ΔΔCT^ formula. The amplification cycles were completed for 40 times and the PCR conditions were as follows: preliminary denaturation at 95˚C for 8 min, followed by 40 cycles of 95˚C for 12 sec, 62˚C for 20 sec and 72˚C for 10 sec. The specificity of reactions was evaluated by way of melting curve examination. The primer sequences were extracted from related literature [3, 22, 32], after blasting and aligning in NCBI (https://www.ncbi.nlm.nih.gov/tools/primer-blast/), was used as approved sequences (Supplementary Table S2).

### Statistical analysis:

SPSS software version 16.0 (IBM Corp., Armonk, NY, USA) was used for data analysis. The independent samples *t*-test and Mann-Whitney *U* test was completed for data analysis and differential gene expression. Receiver operating characteristic (ROC) curve was applied to examine the diagnostic value of lncRNAs. Data were reported as the median or the mean ± SD and p*-*values* <*0.05 were served as statistically significant.

## RESULTS

**Figure 1:**
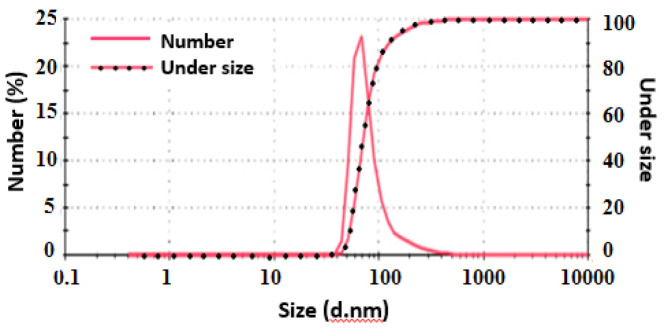
Size detection of exosomes by dynamic light scattering (DLS).

**Figure 2:**
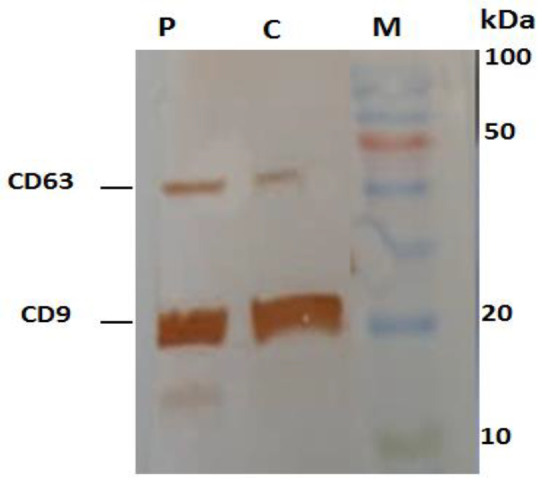
Western blotting analysis of exosomes for CD63 and CD9 proteins.

For confirmation of extracted exosomes from plasma of CRC patients, the size of the exosomal particles was evaluated using DSL. The results verified that the isolated exosomes were 30 to 150 nm in size (Fig. 1). Moreover, characterization of the plasma-derived exosomes was completed by confirming protein markers CD63 and CD9 using Western blotting (Fig. 2).

DLS was used for measurements of exosomes isolated from plasma.

**Figure 3:**
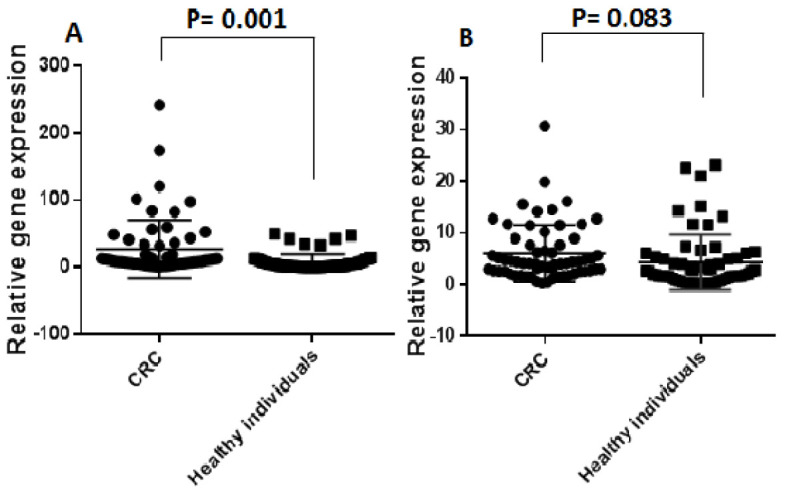
The relative expression levels of the plasma exosomal lncRNAs in 62 CRC patients and 62 healthy people.

The expression pattern of plasma exosomal lncRNAs was assessed by a quantitative real-time PCR in 62 CRC patients and 62 healthy individuals. The results showed that the levels of plasma exosomal lncRNA CCAT1 was meaningfully increased in CRC patients in comparison with those in the healthy people (p=0.001) (Fig. 3). However, the expression level of lncRNA XIST was not significantly change in the plasma-derived exosomes of CRC patients in comparison with those of healthy individuals (p=0.083) (Fig. 3).

Representative Western blotting of DC63 and CD9 markers for exosomes extracted from plasma of CRC patients (P) and healthy individuals as controls (C). M indicates as protein ladder marker (100 kDa).

The expression levels of exosomal lncRNAs CCAT1 (A), but not XIST (B), were dysregulated in CRC patients as compared to the non-cancer persons.

The relationship of exosomal lncRNA CCAT1 expression levels with clinicopathological characterizations of CRC was inspected in 62 CRC patients. It was revealed that the expression levels of exosomal lncRNA CCAT1 was associated with tissue differentiation and lymphovascular invasion (p<0.05). Based on the results, there was no important difference between the mean expression levels of LncRNA XIST and LncRNA CCAT1 and tumor locations in the rectum and colon of patients (p<0.05).

**Figure 4:**
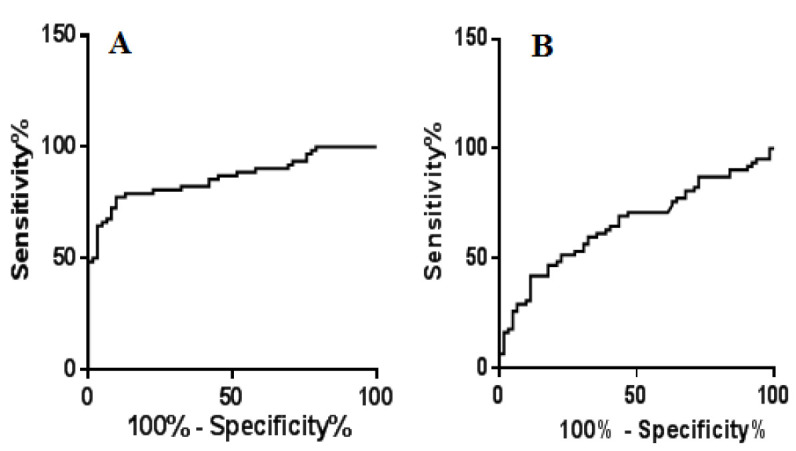
The ROC curves of the plasma exosomal lncRNAs CCAT1 and XIST in CRC.

The ROC analysis was completed for 62 CRC patients to investigate the diagnostic value of exosomal lncRNAs. According to results, it was revealed that the area under the ROC curve (AUC) of exosomal lncRNA CCAT1 was 0.86 and sensitivity 79% and specificity 80%, with a cut off value 6.5 (Fig. 4). The analytic power indicated clinical importance of exosomal lncRNA CCAT1 as potential diagnostic hallmark for patients with CRC. The evaluation of the diagnostic value of lncRNA XIST in CRC using the ROC diagram showed that the lncRNA has had a sensitivity of 62% and a specificity of 61% with a cut off of 2.4, with an AUC=65%, for distinguishing individuals with CRC from healthy controls. The results showed that lncRNA XIST has no a suitable ability to diagnose CRC patients.

A) The AUC of exosomal lncRNA CCAT1 in CRC was 86% with optimal sensitivity 79% and optimal specificity 80%, and a cut off value 6.5. B) The AUC of exosomal lncRNA XIST in CRC was 65% with optimal sensitivity 62% and optimal specificity 61%, and a cut off value 2.4.

## DISCUSSION

The blood-based hallmarks have extensively established as potential non-invasive biomarkers for cancers [33-35]. Various investigations have revealed blood-based exosomal lncRNAs as a potential repertoire for identification of minimally-invasive diagnostic biomarkers for CRC patients. Emerging evidences demonstrated that the circulating and exosome-derived lncRNAs have potentials as diagnostic and prognostic biomarkers for CRC [18-36-37]. Here, we evaluated the expression levels of lncRNAs CCAT1 and XIST in plasma-derived exosomes of CRC patients and investigated their diagnostic values. 

CCAT1 has been documented to upregulate in various clinical samples, including tissue, serum, and plasma, as well CRC cell lines [22, 38]. We showed that the expression of plasma exosomal lncRNA CCAT1 in CRC patients meaningfully differ from healthy individuals. ROC curve analysis revealed a diagnostic power for lncRNA CCAT1 in CRC (sensitivity 79% and specificity 80%, with an AUC=86%). These findings indicated a satisfactory diagnostic value of the exosomal lncRNA CCAT1 in CRC patients. It has been shown that CCAT1 is upregulated by c-Myc (a cancer-driver gene and a hot spot that has genetic alterations). A high level of c-Myc expression was detected in all cases of CCAT1 upregulation [39]. In addition, overexpression of CCAT1 significantly decreased miR155-5p and let7b-5p [40]. Various oncogenic functions and clinical significance of circulating CCAT1 as diagnostic and prognostic biomarker also have been confirmed in a number of malignancies [41-43]. 

Some studies have established the results we observed in dysregulation of the plasma-derived lncRNA in CRC patients [22]. According the results of Abedini et al., the plasma lnc‐CCAT1 (p=0.024) was found to overexpress in the colorectal cancer patients in comparison with the control group. The reported AUC of ROC was 64% (p<0.001), while our ROC curve analysis documented a diagnostic power for exosomal lncRNA CCAT1 in CRC with a sensitivity of 79% and a specificity of 80% with an optimal cutoff point 6.5, with an AUC=86% and p<0.0001. Therefore, most of statistical components, including sensitivity, specificity, and p-value, have improved in our study for exosome-derived lncRNA CCAT1. Hence, according to the comparison of the study results on exosomes and plasma, it can be concluded that the statistical components can be relatively improved in exosomes compared to plasma, which can indicate the advantage of the exosome sample compared to plasma in the assessment of biomarkers. An investigation on tumor tissues of patients with CRC confirmed upregulation of CCAT1 in premalignant conditions and all stages of the disease, including advanced metastatic stage. Based on these data, it was designated that CCAT1 may function in both tumorigenesis and the metastatic processes [44]. 

The clinical data has confirmed lncRNA CCAT1 as a potential candidate for cell-free biopsy assays and an auspicious biomarker for the screening, early detection and management of CRC [45]. Also, some investigations showed that patients with higher tumor stages and poor differentiated tumors have a high expression level of CCAT1 [46]. Our results of the ROC test showed that plasma exosomal lncRNA CCAT1 has a sensitivity of 79%, a specificity of 80%, with an AUC of 86%. In line with these results, Abedini et al. confirmed that CCAT1 had a significant difference between CRC group and healthy individuals with a suitable accuracy, indicating its appropriate discriminatory power for CRC patients [22]. 

It has been documented that the overexpression of XIST in cancer cells may increase cancer invasion and metastasis [47, 48]. Chen et al. found that the upregulation of XIST was correlated with tumor size, grade, metastasis, and TNM stage as well as poor survival of CRC patients [48]. Also, Zheng et al showed that the upregulation of XIST was correlated with the clinical stages and poor survival of CRC patients [49]. Nevertheless, there is little research on the plasma investigation of EVs for evaluating lncRNA XIST expression and clinical potentials. Yu et al. showed that the expression of lncRNA XIST in the extracellular vesicles of the serum of CRC patients is significantly increased [50]. 

In addition to CRC, Lan et al. showed a noteworthy increase in tumor tissue and serum exosomal XIST of triple negative breast cancer (TNBC) patients [31]. In agreement with our study, they reported no relationship with clinical and pathological parameters such as tumor size, differentiation and invasion. Our results showed that the expression of lncRNA XIST did not considerably change in exosomes derived from the plasma of patients with CRC. In opposite with the present results, Yu confirmed that serum vesicular XIST has a sensitivity of 88.3%, specificity of 90.2%, and AUC=86.4%, which indicates its appropriate discrimination power [50]. Among the reasons for these variations, we can notice the number of clinical samples studied and the type or origin of the samples. Some studies have reported significant dysregulation of these non-coding RNAs in tissue- and blood-based samples, while the expression pattern of these molecules in exosomes may be obviously different. Also, the levels and pattern of their expression may be varied in different cancers, especially in samples with different clinical origins.

## Conflict of Interest:

The authors declare no conflicts of interest.

## Authors’ Contribution:

FD, AA, SM contributed to study conceptualization, data curation and project administration. AA and SM completed funding acquisition and resources. FD, ZSE and AN contributed in methodology, data curation and formal data analysis. FD, AA, SPT contributed in clinical investigation, supervision, validation and visualization. AA and FD prepared the original draft or critical revision for important intellectual content. All authors approved the final version of the draft.

## References

[1] Siegel RL, Miller KD, Wagle NS, Jemal A (2023). Cancer statistics, 2023. CA Cancer J Clin.

[2] Cardenas C, Abrishami S (2023). Colorectal carcinoma: Review and novelties in 2023. Clin Oncol Case Rep.

[3] Karamzadeh AR, Heidari M, Namazi A, Tabaeian SP, Akbari A (2024). The dysregulation and clinical relevance of lncRNAs MYOSLID and SFTA1P in colorectal cancer patients. Mol Biol Rep.

[4] Akbari A, Mobini GR, Maghsoudi R, Akhtari J, Faghihloo E, Farahnejad Z (2016). Modulation of transforming growth factor‑β signaling transducers in colon adenocarcinoma cells induced by staphylococcal enterotoxin B. Mol Med Rep.

[5] Tepus M, Yau TO (2020). Non-invasive colorectal cancer screening: an overview. Gastrointest Tumors.

[6] Ferrari A, Neefs I, Hoeck S, Peeters M, Van Hal G (2021). Towards novel non-invasive colorectal cancer screening methods: a comprehensive review. Cancers (Basel).

[7] Tabaeian SP, Eshkiki ZS, Dana F, Fayyaz F, Baniasadi M, Agah S, Masoodi M, Safari E, Sedaghat M, Abedini P, Akbari A (2024). Evaluation of tumor-educated platelet long non-coding RNAs (lncRNAs) as potential diagnostic biomarkers for colorectal cancer. J Cancer Res Ther.

[8] Agah S, Akbari A, Talebi A, Masoudi M, Sarveazad A, Mirzaei A, Nazmi F (2017). Quantification of plasma cell-free circulating DNA at different stages of colorectal cancer. Cancer Invest.

[9] Molnár B, Galamb O, Kalmár A, Barták BK, Nagy ZB, Tóth K, Tulassay Z, Igaz P, Dank M (2019). Circulating cell-free nucleic acids as biomarkers in colorectal cancer screening and diagnosis-an update. Expert Rev Mol Diagn.

[10] Toth K, Bartak BK, Tulassay Z, Molnar B (2016). Circulating cell-free nucleic acids as biomarkers in colorectal cancer screening and diagnosis. Expert Rev Mol Diagn.

[11] Thakur K, Singh MS, Feldstein-Davydova S, Hannes V, Hershkovitz D, Tsuriel S (2021). Extracellular vesicle-derived DNA vs cfDNA as a biomarker for the detection of colon cancer. Genes (Basel).

[12] Ruivo CF, Adem B, Silva M, Melo SA (2017). The biology of cancer exosomes: insights and new perspectives. Cancer Res.

[13] Kalluri R (2016). The biology and function of exosomes in cancer. J Clin Invest.

[14] Kok VC, Yu CC (2020). Cancer-derived exosomes: their role in cancer biology and biomarker development. Int J Nanomedicine.

[15] Boriachek K, Islam MN, Möller A, Salomon C, Nguyen NT, Hossain MSA, Yamauchi Y, Shiddiky MJA (2018). Biological functions and current advances in isolation and detection strategies for exosome nanovesicles. Small.

[16] LeBleu VS, Kalluri R (2020). Exosomes as a multicomponent biomarker platform in cancer. Trends Cancer.

[17] Hon KW, Abu N, Ab Mutalib NS, Jamal R (2017). Exosomes as potential biomarkers and targeted therapy in colorectal cancer: a mini-review. Front Pharmacol.

[18] Baassiri A, Nassar F, Mukherji D, Shamseddine A, Nasr R, Temraz S (2020). Exosomal non coding RNA in LIQUID biopsies as a promising biomarker for colorectal cancer. Int J Mol Sci.

[19] Wu Q, Liu W, Wang J, Zhu L, Wang Z, Peng Y (2020). Exosomal noncoding RNAs in colorectal cancer. Cancer Lett.

[20] Deng H, Wang JM, Li M, Tang R, Tang K, Su Y, Hou Y, Zhang J (2017). Long non-coding RNAs: New biomarkers for prognosis and diagnosis of colon cancer. Tumour Biol.

[21] Saus E, Brunet-Vega A, Iraola-Guzman S, Pegueroles C, Gabaldon T, Pericay C (2016). Long non-coding RNAs as potential novel prognostic biomarkers in colorectal cancer. Front Genet.

[22] Abedini P, Fattahi A, Agah S, Talebi A, Beygi AH, Amini SM, Mirzaei A, Akbari A (2019). Expression analysis of circulating plasma long noncoding RNAs in colorectal cancer: The relevance of lncRNAs ATB and CCAT1 as potential clinical hallmarks. J Cell Physiol.

[23] Talebi A, Azizpour M, Agah S, Masoodi M, Mobini GR, Akbari A (2020). The relevance of long noncoding RNAs in colorectal cancer biology and clinical settings. J Cancer Res Ther.

[24] Akbari A, Abbasi S, Borumandnia N, Eshkiki ZS, Sedaghat M, Tabaeian SP, Faghihi Kashani A, Talebi A (2022). Epigenetic regulation of gastrointestinal cancers mediated by long non-coding RNA. Cancer Biomark.

[25] Zhao Y, Song X, Song X, Xie L (2022). Identification of Diagnostic Exosomal LncRNA-miRNA-mRNA Biomarkers in Colorectal Cancer Based on the ceRNA Network. Pathol Oncol Res.

[26] Xiao Y, Zhong J, Zhong B, Huang J, Jiang L, Jiang Y, Yuan J, Sun J, Dai L, Yang C, Li Z, Wang J, Zhong T (2020). Exosomes as potential sources of biomarkers in colorectal cancer. Cancer Lett.

[27] Liau XL, Salvamani S, Gunasekaran B, Chellappan DK, Rhodes A, Ulaganathan V, Tiong YL (2023). CCAT 1-a pivotal oncogenic long non-coding RNA in colorectal cancer. Br J Biomed Sci.

[28] Nissan A, Stojadinovic A, Mitrani‐Rosenbaum S, Halle D, Grinbaum R, Roistacher M, Bochem A, Dayanc BE, Ritter G, Gomceli I, Bostanci EB, Akoglu M, Chen YT, Old LJ, Gure AO (2012). Colon cancer associated transcript‐1: a novel RNA expressed in malignant and pre‐malignant human tissues. Int J Cancer.

[29] Wang L, Cho KB, Li Y, Tao G, Xie Z, Guo B (2019). Long noncoding RNA (lncRNA)-mediated competing endogenous RNA networks provide novel potential biomarkers and therapeutic targets for colorectal cancer. Int J Mol Sci.

[30] Dastmalchi N, Safaralizadeh R, Nargesi MM (2020). LncRNAs: potential novel prognostic and diagnostic biomarkers in colorectal cancer. Curr Med Chem.

[31] Lan F, Zhang X, Li H, Yue X, Sun Q (2021). Serum exosomal lncRNA XIST is a potential non‐invasive biomarker to diagnose recurrence of triple‐negative breast cancer. J Cell Mol Med.

[32] Omrani Tabarestani F, Akbari A, Zare Karizi S, Sotoodehnejadnematalahi F (2022). Regulation of long non-coding RNAs XIST and ROR induced by homeodomain protein TGIF2LX in colorectal cancer. J Cancer Res Ther.

[33] Crotti S, Agnoletto E, Cancemi G, Di Marco V, Traldi P, Pucciarelli S, Nitti D, Agostini M (2016). Altered plasma levels of decanoic acid in colorectal cancer as a new diagnostic biomarker. Anal Bioanal Chem.

[34] Zygulska AL, Pierzchalski P (2022). Novel diagnostic biomarkers in colorectal cancer. Int J Mol Sci.

[35] Hauptman N, Glavač D (2017). Colorectal cancer blood-based biomarkers. Gastroenterol Res Pract.

[36] Hu D, Zhan Y, Zhu K, Bai M, Han J, Si Y, Zhang H, Kong D (2018). Plasma exosomal long non-coding RNAs serve as biomarkers for early detection of colorectal cancer. Cell Physiol Biochem.

[37] Rizk NI, Abulsoud AI, Kamal MM, Kassem DH, Hamdy NM (2022). Exosomal-long non-coding RNAs journey in colorectal cancer: Evil and goodness faces of key players. Life Sci.

[38] Ye Z, Zhou M, Tian B, Wu B, Li J (2015). Expression of lncRNA-CCAT1, E-cadherin and N-cadherin in colorectal cancer and its clinical significance. Int J Clin Exp Med.

[39] He X, Tan X, Wang X, Jin H, Liu L, Ma L, Yu H, Fan Z (2014). C-Myc-activated long noncoding RNA CCAT1 promotes colon cancer cell proliferation and invasion. Tumour Biol.

[40] Arunkumar G, Murugan AK, Prasanna Srinivasa Rao H, Subbiah S, Rajaraman R, Munirajan AK (2017). Long non-coding RNA CCAT1 is overexpressed in oral squamous cell carcinomas and predicts poor prognosis. Biomed Rep.

[41] Xiao K, Dong Z, Wang D, Liu M, Ding J, Chen W, Shang Z, Yue C, Zhang Y (2021). Clinical value of lncRNA CCAT1 in serum extracellular vesicles as a potential biomarker for gastric cancer. Oncol Lett.

[42] Zhang XF, Liu T, Li Y, Li S (2015). Overexpression of long non-coding RNA CCAT1 is a novel biomarker of poor prognosis in patients with breast cancer. Int J Clin Exp Pathol.

[43] Jiang X, Li ZL, Li JL, Zheng WY, Li XH, Cui YF, Sun DJ (2017). LncRNA CCAT1 as the unfavorable prognostic biomarker for cholangiocarcinoma. Eur Rev Med Pharmacol Sci.

[44] Zong D, Liu X, Li J, Long Y, Ouyang R, Chen Y (2022). LncRNA-CCAT1/miR-152-3p is involved in CSE-induced inflammation in HBE cells via regulating ERK signaling pathway. Int Immunopharmacol.

[45] Gharib E, Nazemalhosseini‐Mojarad E, Baghdar K, Nayeri Z, Sadeghi H, Rezasoltani S, Jamshidi-Fard A, Larki P, Sadeghi A, Hashemi M, Asadzadeh Aghdaei H (2021). Identification of a stool long non‐coding RNAs panel as a potential biomarker for early detection of colorectal cancer. J Clin Lab Anal.

[46] Iranmanesh H, Entezari M, Rejali L, Nazemalhosseini-Mojarad E, Maghsoudloo M, Asadzadeh Aghdaei H, Zali MR, Hushmandi K, Rabiee N, Makvandi P, Ashrafizadeh M, Hashemi M (2022). The association of clinicopathological characterizations of colorectal cancer with membrane-bound mucins genes and LncRNAs. Pathol Res Pract.

[47] Li C, Wan L, Liu Z, Xu G, Wang S, Su Z, Zhang Y, Zhang C, Liu X, Lei Z, Zhang HT (2018). Long non-coding RNA XIST promotes TGF-β-induced epithelial-mesenchymal transition by regulating miR-367/141-ZEB2 axis in non-small-cell lung cancer. Cancer Lett.

[48] Chen DL, Chen LZ, Lu YX, Zhang DS, Zeng ZL, Pan ZZ, Huang P, Wang FH, Li YH, Ju HQ, Xu RH (2017). Long noncoding RNA XIST expedites metastasis and modulates epithelial–mesenchymal transition in colorectal cancer. Cell Death Dis.

[49] Zheng H, Zhang M, Ke X, Deng X, Li D, Wang Q, Yan S, Xue Y, Wang Q (2021). LncRNA XIST/miR-137 axis strengthens chemo-resistance and glycolysis of colorectal cancer cells by hindering transformation from PKM2 to PKM1. Cancer Biomark.

[50] Yu J, Dong W, Liang J (2020). Extracellular vesicle-transported long non-coding RNA (LncRNA) X inactive-specific transcript (XIST) in serum is a potential novel biomarker for colorectal cancer diagnosis. Med Sci Monit.

