# Supplementary material for: Clinical relevance of plasma-derived exosomal long non-coding RNAs (lncRNAs) CCAT1 and XIST in colorectal cancer patients
Source: Mol Biol Res Commun. 2025;14(2):157–66. doi: 10.22099/mbrc.2025.51654.2061 (PMC11865934; doi:10.22099/mbrc.2025.51654.2061)
Supplement: Supplementary file 1 [file MBRC-14-157-s001.pdf]

Table S1: Oligonucleotide sequences used as specific primers for lncRNAs and internal control.

**Gene/lncRNA**	**Primer sequence**	Reference
β -Actin	Forward: 5´-AGAGCTACGAGCTGCCTGAC-3´	[3]
	Reverse: 5´-AGCACTGTGTTGGCGTACAG-3´	
Lnc-XIST	Forward: 5´-TGACCTTGTTAAGCAAGCG-3´	[32]
	Reverse: 5´-ATGGACCACTGTTTGATAGAC-3´	
Lnc-CCAT1	Forward: 5´- TTTATGCTTGAGCCTTGA-3´	[22]
	Reverse: 5´- CTTGCCTGAAATACTTGC-3´	

Table S2: The relationship between demographic and clinicopathological variables and the expression levels of CCAT1 and XIST in CRC patients.

**Parameters**		***CCAT1 (p-value)***	***XSIT (p-value)***
**Age**	<60 (n=27)	0.42	0.9
	≥60 (n=35)		
**Sex**	Male (n=41)	0.52	0.36
	Female (n=21)		
**Location**	Colon (n=15)	0.41	0.86
	Rectum (n=47)		
**Polyp**	Positive (n=15)<br>Negative (n=47)	0.25	0.4
**Colitis**	Positive (n=33)<br>Negative (n=29)	0.13	0.6
**Metastasis**	Positive (n=19)<br>Negative (n=43)	0.82	0.53
**Pathology**	Well (n=46)		
	Moderate (n=11)	0.33	0.54
	Poor (n=5)		
**Genetic history**	Positive (n=9)<br>Negative (n=53)	0.8	0.44
**Tumor size**	<2 cm (n=15)		
	2-3.99 cm (n=36)	0.28	0.3
	≥ 4 cm (n=11)		
**Tumor stage**	T2 (n=38)		
	T3 (n=17)<br>T4 (n=7)	0.35	0.16
